# Supplementary material for: Surface enrichment and diffusion enabling gradient-doping and coating of Ni-rich cathode toward Li-ion batteries
Source: Nat Commun. 2021 Jul 27;12:4564. doi: 10.1038/s41467-021-24893-0 (PMC8316340; doi:10.1038/s41467-021-24893-0)
Supplement: Supplementary file 1 — Supplementary Information [file 41467_2021_24893_MOESM1_ESM.pdf]

Supplementary Information

Surface enrichment and diffusion enabling gradient-doping and coating of Ni-rich cathode toward Li-ion batteries

Haifeng Yu[1,4], Yueqiang Cao[2,4], Long Chen[1], Yanjie Hu[1], Xuezhi Duan[2], Sheng Dai[3], Chunzhong Li[1], Hao Jiang[1]*

[1]Key Laboratory for Ultrafine Materials of Ministry of Education, Shanghai Engineering Research Center of Hierarchical Nanomaterials, School of Materials Science and Engineering, East China University of Science and Technology, Shanghai 200237, China

[2]State Key Laboratory of Chemical Engineering, School of Chemical Engineering, East China University of Science and Technology, Shanghai 200237, China

[3]Key Laboratory for Advanced Materials and Feringa Nobel Prize Scientist Joint Research Center, School of Chemistry & Molecular Engineering, East China University of Science and Technology, Shanghai 200237, China

[4]These authors contributed equally: Haifeng Yu, Yueqiang Cao

*Corresponding author. Email: jianghao@ecust.edu.cn (Prof. H. Jiang)

Tel.: +86-21-64250949, Fax: +86-21-64250624

Supplementary Note 1. Method and model of finite element analysis

The coupled mechanical and thermal modules in finite element analysis were carried to obtain the volume deformation and internal stress of cathodes at different stage of charge. The elastic modulus, Poisson's ratio and density of the material were set to 116 GPa, 0.25 and 2.75 g cm^{-3}, respectively. The initial displacement and speed were both set to 0 and the boundary conditions were fixed constraint boundary conditions. The relative tolerance of convergence

condition was sat as 0.001. The grain orientation was random (in the range of 0-360 °). The transformation matrix was used to represent the relationship between the local expansion/contraction and the global expansion/contraction: $\alpha_{ij}^{L} = tr_{ki} tr_{lj} \alpha_{kl}$. The corresponding matrix was shown below.

$$\begin{pmatrix} \cos[\alpha]\cos[\theta]-\cos[\beta]\sin[\alpha]\sin[\theta] & \sin[\alpha]\cos[\theta]+\cos[\beta]\cos[\alpha]\sin[\theta] & \sin[\beta]\sin[\theta] \\ -\cos[\alpha]\cos[\theta]\sin[\alpha]-\cos[\alpha]\sin[\theta] & \cos[\beta]\cos[\alpha]\cos[\theta]-\sin[\alpha]\sin[\theta] & \sin[\beta]\cos[\theta] \\ \sin[\alpha]\sin[\beta] & -\cos[\alpha]\sin[\beta] & \cos[\beta] \end{pmatrix}$$

Supplementary Note 2. The testing process and calculation equation of GITT measurement

Before the GITT measurement, the cells were firstly galvanostatic charge/discharged for 5 cycles. GITT measurements were performed by charging/discharging the fully activated cells at a constant current (0.1 C) for an interval of 20 min followed by an open circuit stand for 2 h to allow the cell voltage to relax to its quasi-equilibrium state. The change in the steady-state voltage $\triangle E_s$ is obtained by subtracting the original voltage (E_0) from the steady-state voltage (E_s). The cell voltage increases during the current flux and the total change of cell voltage ΔE_τ can be obtained by calculating the voltage drop. Meanwhile, the process of the chemical diffusion is assumed to obey Fick's second law of diffusion. With a series of simplifications, for sufficient time interval ($\tau \ll L^2/D_{Li+}$), the equation of D_{Li+} can be written as (Equation 2):

$$D_{Li^+} = \frac{4}{\pi}\left(\frac{m_B V_m}{M_B A}\right)^2 \left(\frac{\triangle E_s}{\tau\left(\frac{dE_\tau}{d\sqrt{\tau}}\right)}\right)^2 \ (\tau << \frac{L^2}{D_{Li}{}^+}) \qquad (2)$$

where V_m is the molar volume of active materials, M_B and m_B are the molecular weight and mass of the host oxide, respectively, and A is the total contact area between the electrolyte and the electrode, L is the thickness of the electrode. If sufficiently small currents and short time

intervals are employed, the cell voltage is a linear function of the square root of τ, Equation 2 can be further simplified as (Equation 3):

$$D_{Li^+} = \frac{4L^2}{\pi\tau}\left(\frac{\triangle E_S}{\triangle E_\tau}\right)^2 \quad (\tau << \frac{L^2}{D_{Li^+}}) \qquad (3)$$

Supplementary Note 3. The testing process of fast charge/discharge capabilities

Fast charge capacities: The cells were charged by CC (constant current)- CV (constant voltage) program. In detail, the cell was first charged to 4.3 V at different current density from 0.5 C and 5 C. Then, the cell was CV charged at voltage of 4.3 V until the current reduces to 0.05 C. Besides, the charge time also needs to set the upper limit according to the charging current. Finally, the cells were discharged at the same current density of 1 C to compare the capacity.

Fast discharge capacities: The cells were all charged by CC (constant current)- CV (constant voltage) program. The cell was first charged to 4.3 V at 1 C, and the cell was CV charged at voltage of 4.3 V until the current reduces to 0.05 C. Finally, the cells were discharged at the different current density from 0.5 C to 5 C, which can compare the capability of high-power discharge.

Supplementary Figures

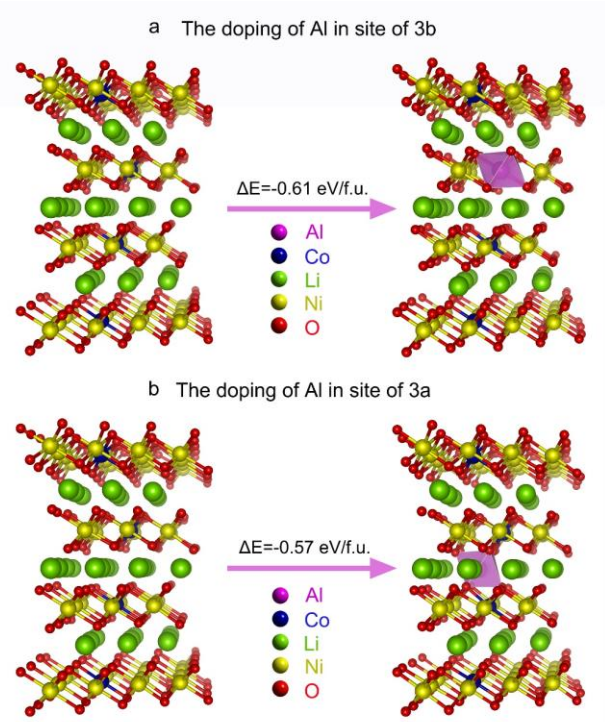

Supplementary Fig. 1 Atomic configurations of Al-doped at (a) Ni site and (b) Li site with corresponding formation energy.

The formation energies are compared for two possible doping sites (Ni sites and Li sites) in the lattice of Ni-rich cathode to determine the most energetically preferable dopant location of Al^{3+}. As shown in **Supplementary Fig. 1** and **Supplementary Table 1**, the formation energy of Al^{3+} at a Ni-site is lower than Li-site. Therefore, the Al^{3+} will locate at 3b sites of Ni^{2+} in Al-doped Ni-rich cathodes lattice.

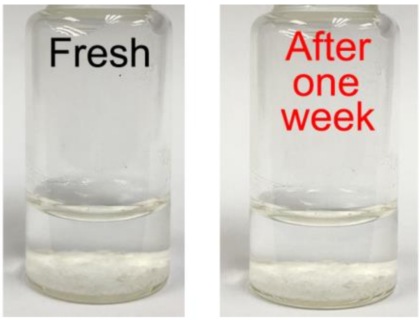

Supplementary Fig. 2 The digital photograph of $LiAlO_2$ immersed within the electrolyte after one week.

To check the chemical stability of $LiAlO_2$ in organic electrolyte, 200 mg of $LiAlO_2$ was immersed within 3 mL of electrolyte for one week, and the amount of Al in the supernatant was determined by ICP-OES. No dissolved Al element was detected by ICP-OES, which is matched well with clear and transparent supernatant in digital photograph (**Supplementary Fig. 2**).

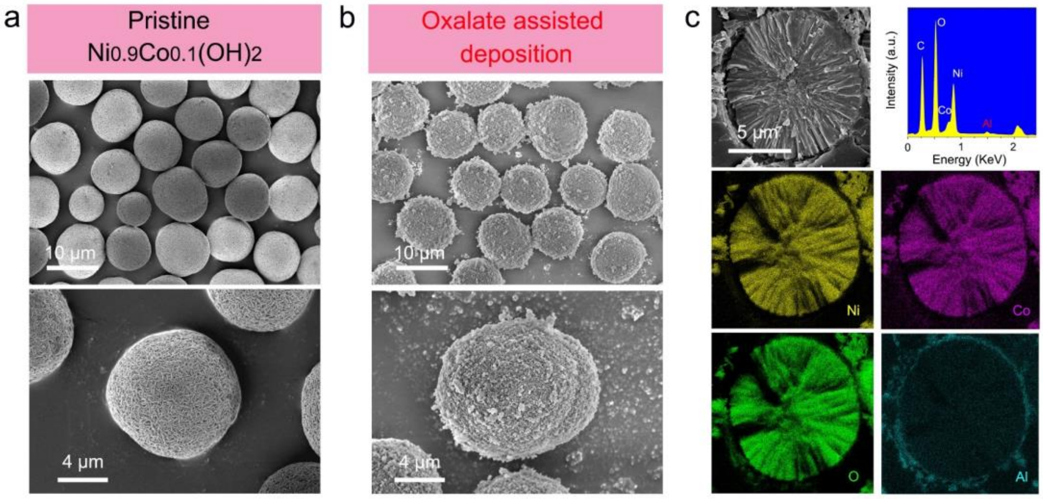

Supplementary Fig. 3 The SEM images of (a) the $Ni_{0.9}Co_{0.1}(OH)_2$ precursors and (b) the NC91-$Al(OH)_3$ precursors with oxalate-assisted deposition. (c) The cross-sectional SEM image of the NC91-$Al(OH)_3$ precursors with EDS spectrum and mapping images of Ni, Co, O and Al elements.

The $Al(OH)_3$ coated Ni-rich precursors were prepared by the methods of oxalate-assisted deposition. Compared to the pristine precursors, the uniform coating layer of $Al(OH)_3$ exists on the surface, which is demostrated by EDS mapping images.

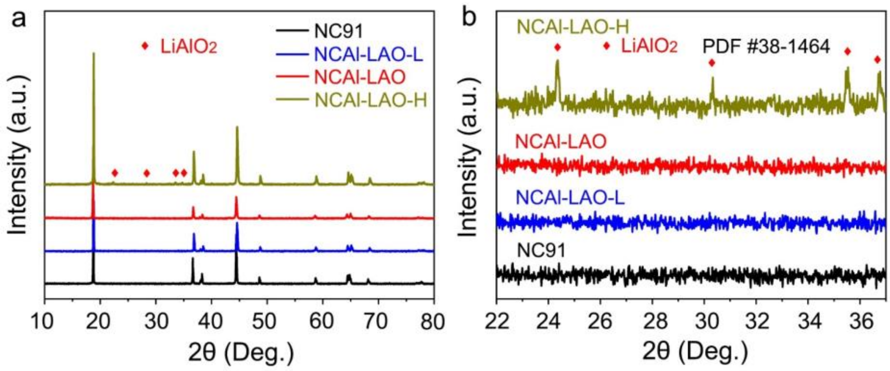

Supplementary Fig. 4 (a, b) XRD patterns of the samples with partially enlarged XRD patterns.

The modified Ni-rich cathodes with various aluminum content were prepared. As shown in **Supplementary Table 4**, the relative fraction of Ni, Co, and Al are well consistent with our purported molar ratio according to the inductively coupled plasma atomic emission spectroscopy (ICP-AES). **Supplementary Fig. 4** displays the XRD patterns of the samples with various aluminum content. The main diffraction peaks are all assignable to the space group of R-3m (no. 166) and well-matched with the hexagonal $LiNiO_2$ (JCPDS 09-0063). The distinct splitting of (006)/(012) and (018)/(110) peaks imply an ordered layered structure, manifesting that the bulk structures of Ni-rich oxide are not destroyed after modification process[1]. With the increasement of the $Al(OH)_3$ content, some other diffraction peaks matched with $LiAlO_2$ (JCPDS 38-1464) arise in XRD pattern of NCAl-LAO-H[2]. It is because that the coating layer of $Al(OH)_3$ on the surface will react with LiOH and *in situ* transform to $LiAlO_2$ during calcination process. Besides, the relatively low content of $LiAlO_2$ in NCAl-LAO-L and NCAl-LAO cause that no peaks corresponding to $LiAlO_2$ can be observed. The corresponding values of $I_{(003)}/I_{(104)}$ are also calculated and listed in **Supplementary Table 5**, which can reflect the degree of Li/Ni disorder[1]. It is noted that the values gradually enhance with the increasement of Al element, suggesting that the Al^{3+} is capable for moderating the Li/Ni disorder. However,

it is noticed that overmuch Al element will generate excess electrochemical inert $LiAlO_2$, which will dramatically reduce the specific capacity of the cathode. Therefore, synergistically considering the specific capacity and comprehensive electrochemical properties, it is logical to speculate that NCAl-LAO is the most suitable cathode in the series of materials for this work, which is selected to deeply scrutinize.

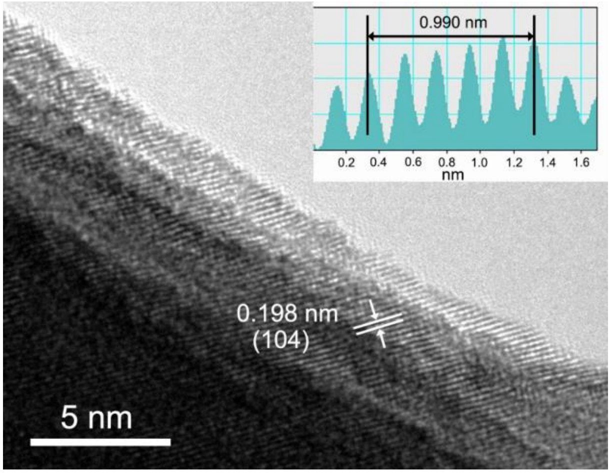

Supplementary Fig. 5 HRTEM image of NC91.

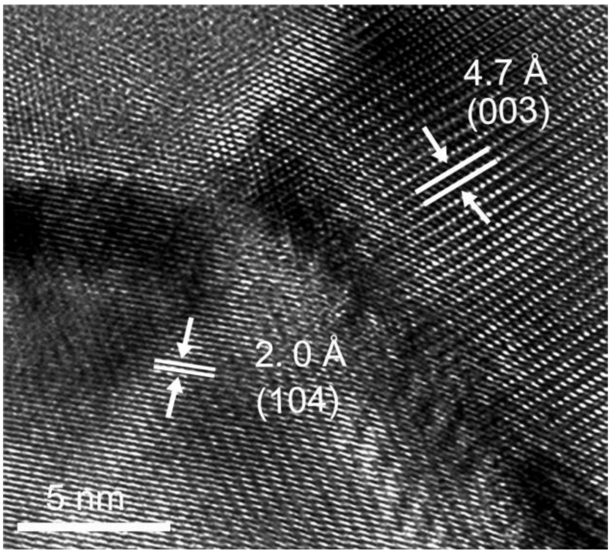

Supplementary Fig. 6 High-resolution TEM images of interior for NCAl-LAO

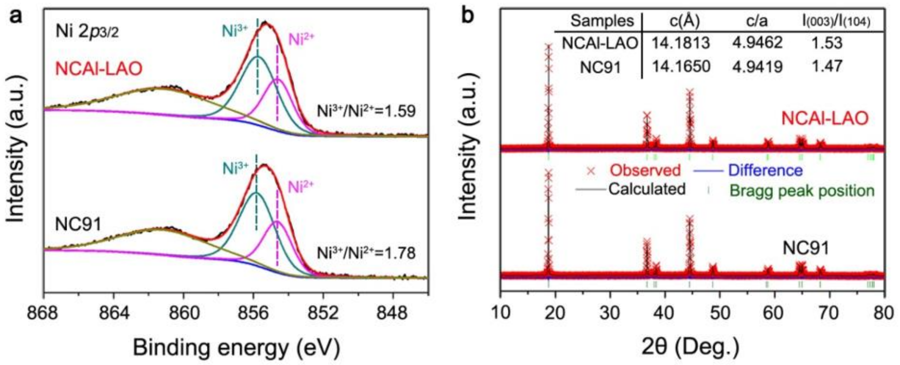

Supplementary Fig. 7 (a) Ni 2p XPS spectra and (b) XRD Rietveld refinements of NCAl-LAO and NC91.

The change of chemical composition and element valence after modification was measured via XPS characterization. The detailed analysis of Ni2$p_{3/2}$ XPS spectra were exhibited in **Supplementary Fig. 7a**. The specific signals of Ni 2$p_{3/2}$ situated at 854.6 and 855.9 eV clearly affirm the coexistence of Ni^{2+} and Ni^{3+} in the samples[3]. Intriguingly, the peak area ratio of Ni^{3+}/Ni^{2+} for NCAl-LAO (1.59) is smaller than that of NC91 (1.78), which is because that Ni^{3+} are partly reduced after the incorporation of Al^{3+} to maintain the charge balance. The XRD Rietveld refinements were actualized to further disclose atomic occupancy and the lattice parameters, and the patterns with corresponding data were exhibited in **Supplementary Fig. 7b** and **Supplementary Table 6**. The low values of R_{wp} and χ^2 confirm that the refinements are valid. The Al^{3+} in NCAl-LAO occupy the 3b sites in transition-metal (TM) layer. Compared with the NC91, the lattice parameters (a, c) and the unit volume of NCAl-LAO slightly amplify, which is attributed to the increased content of Ni^{2+} with larger ionic radius. Impressively, the NCAl-LAO with more Ni^{2+} still displays a lower value of Li/Ni disorder (1.3%) than that of NC91 (2.4%), which is also verified by the larger c/a. Conceivably, the NCAl-LAO with the larger lattice parameters and the lower Li/Ni disorder will display higher intrinsic Li^+

conductivity and more stable crystal structure during lithiation/delithiation process.

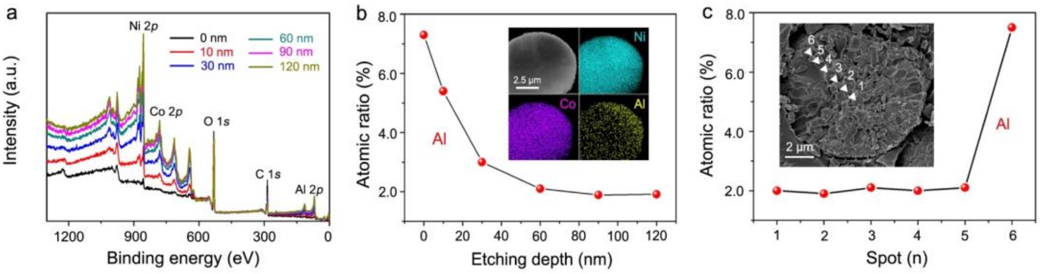

Supplementary Fig. 8 (a) XPS spectra of NCAl-LAO with various argon ion etching depths. (b) Relative content of Al as a function of etching depth based on XPS of NCAl-LAO, inset in (b) shows STEM-EDS element mapping. (c) The Al^{3+} distribution across the secondary particles of NCAl-LAO detected by EDS point analyses. The white triangles (numbers are indicated) mark different point for EDS analysis in a single microsphere.

To further certify the Al^{3+}doping throughout NCAl-LAO cathodes, EDS point analyses in a cross-section of single secondary microsphere were carried out to scrutinize the atomic ratio of Al^{3+} at different areas, and the data was displayed in **Supplementary Fig. 8c**. It is noted that the content of Al^{3+} on the surface of secondary particles is extraordinarily high, which is due to the coating of $LiAlO_2$ on the surface. The similar atomic ratio of approximately 2% at other internal areas indicates that the Al^{3+} distribute over all the area in secondary particles.

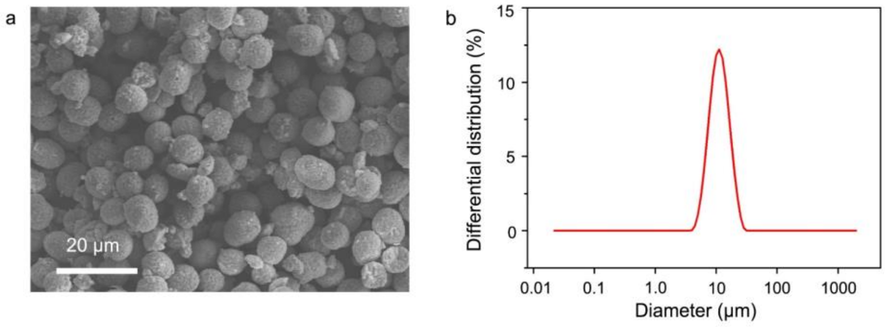

Supplementary Fig. 9 (a) SEM image and (b) the distributions of particle size for NCAl-LAO.

The low-magnification field emission scanning electron microscopy (FESEM) image of NCAl-LAO displays typical spherical shape consisted small primary particles with sizes of ~400 nm, which means that the modification process has little destruction for microsphere structure. The median diameter (D_{50}) in the size distribution diagram of the NCAl-LAO is 10.5 μm, and the span of the particle size (calculated by $(D_{90}-D_{10})/D_{50}$) is 0.965[1,4], implying a typical normal distribution of the dimension for NCAl-LAO microspheres.

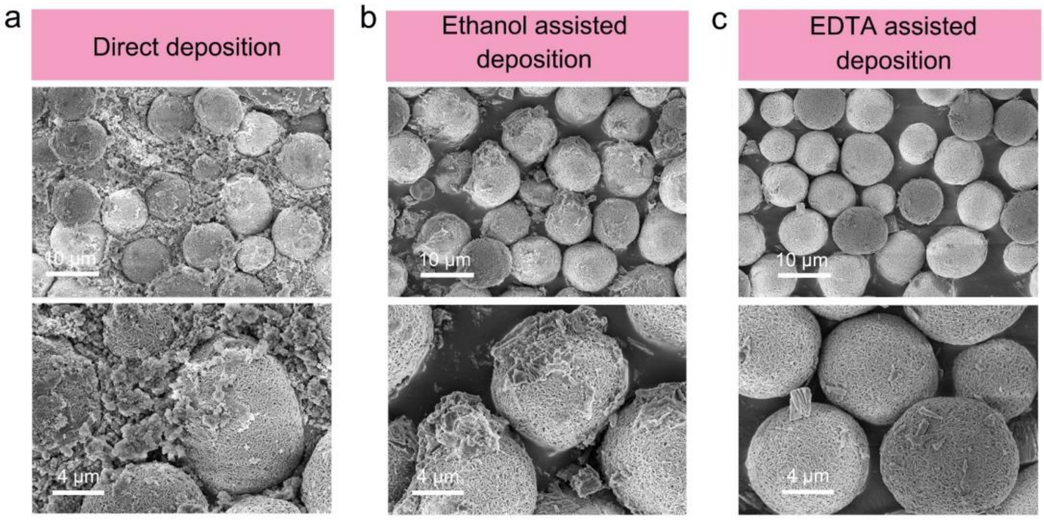

Supplementary Fig. 10 The images of coated precursors (a) without ligand, with ligand of (b) ethanol and (c) EDTA, respectively.

The modified precursors without ligand and with ethanol or EDTA ligand were also prepared and the corresponding FESEM images were exhibited in **Supplementary Fig. 10**. As predicted by theoretical calculation, the $Al(OH)_3$ did not form uniform coating during the deposition process without ligand and with ethanol ligand. When the EDTA is used as ligand, there is almost no substance on the surface of the particles. The STEM and XRD results clearly certify the oxalate-assisted modification process.

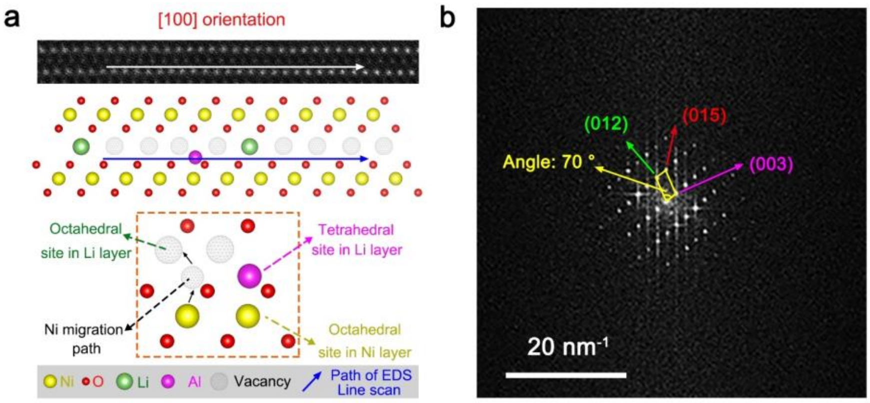

Supplementary Fig. 11 (a) The crystal structure of Ni-rich cathode in the [100] orientation and the schematic diagram of EDS line scan. (b) The FFT pattern of NCAl-LAO obtained from HAADF image in **Fig 2e**.

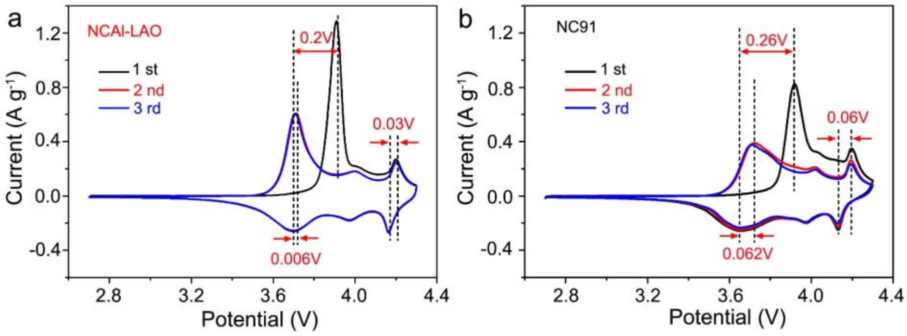

Supplementary Fig. 12 The initial three CV curves of NCAl-LAO and NC91.

There are three couple peaks located at 3.75/3.66, 4.01/3.95, 4.20/4.15 V vs. Li/Li^+, respectively. They are corresponding to the three phase transition processes: H1 (hexagonal phase) → M (monoclinic phase), M (the monoclinicphase) → H2 (hexagonal phase), and H2 (hexagonal phase) → H3 (hexagonal phase)[3]. The potential intervals (ΔE) between anodic and cathodic peaks in the CV curves can reflect the electrode polarization[5]. For the H1-M phase transition in first curves, the ΔE of NCAl-LAO (0.2 V) is lower than that of NC91 (0.26 V). The ΔE of NCAl-LAO at H1-M and H2-H3 phase transition during the subsequent secondary cycles are 0.006 and 0.03 eV, whilst the correpnding values in NC91 are up to 0.062 and 0.06 eV.

Therefore, the electrode polarization is effectively moderated after modification, which is consistent well with enhanced rate performance. Moreover, the better coincidence of NCAl-LAO in the secondary and third cycles further demonstrates a preferable reversibility during the lithiation/delithiation process.

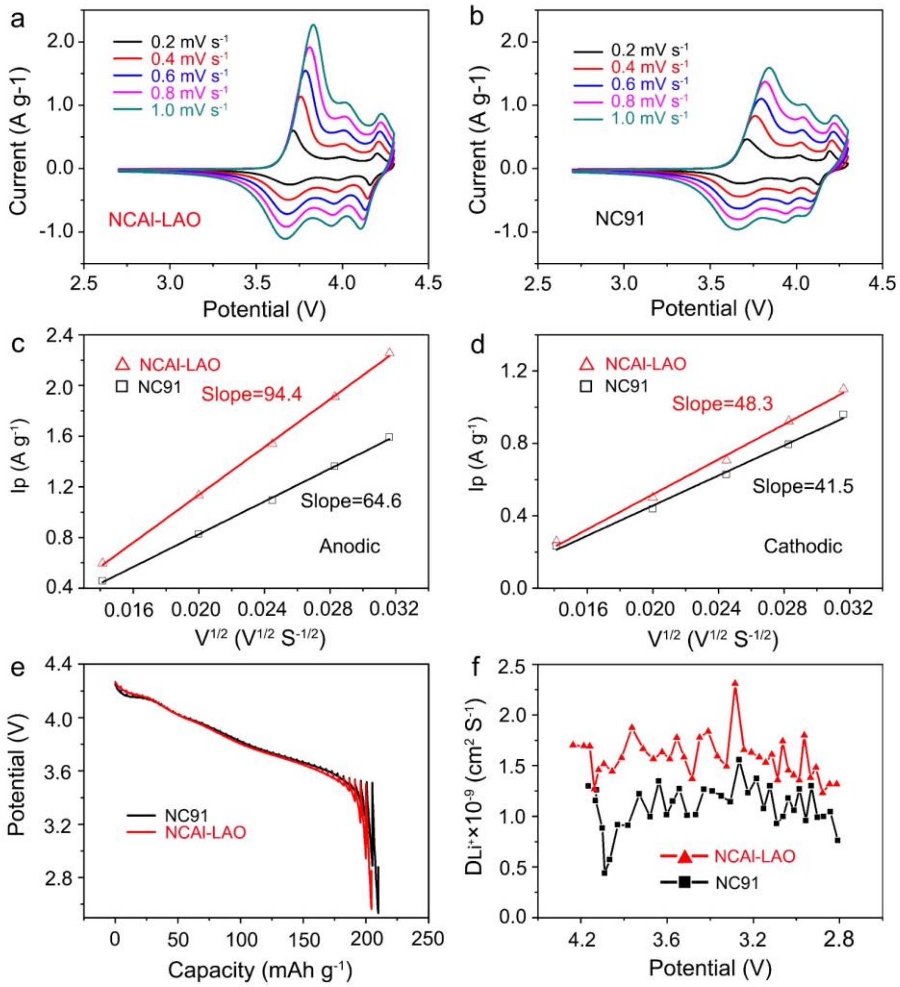

Supplementary Fig. 13 CV curves of (a) NCAl-LAO and (b) NC91 from 0.2 to 1mV s^{-1}. (c) Linear relationship between the anodic/cathodic peak current (i_p) and the square root of the scan rate ($v^{1/2}$) for NCAl-LAO and NC91. GITT of (e) discharge curves and (f) Li^+ diffusion coefficient calculated by GITT for NCAl-LAO and NC91.

The CV curves were obtained by operating at various scan rates of 0.2, 0.4, 0.6, 0.8 and 1 mV

s^{-1}, respectively (**Supplementary Fig. 13a, b**). The fine linear relationship between the peak current (i_p) and the square root of the scanning rate ($v^{1/2}$) indicates the Li^+ transfer in these cathodes exhibits a diffusion-controlled process, and the larger slopes of NCAl-LAO demonstrate higher diffusivity than that in NC91 (**Supplementary Fig. 13c, d)**[3,6]. The GITT analysis was also carried out to obtain the specific Li^+ diffusion coefficients at each stage of cell operation. **Supplementary Fig. 13e** shows the GITT curves of NCAl-LAO and NC91 in discharging process, while the testing process and calculation equation are presented in **Supplementary notes**. The as-calculated Li^+ diffusion coefficients (D_{Li+}) as a function of the Li extraction content are shown in **Supplementary Fig. 13f**. The values of the two samples are both 10^{-10}-10^{-9} cm^2 s^{-1}, which is in accordance with the earlier reports[3,7]. Impressively, the NCAl-LAO displays higher Li^+ diffusion coefficients than that of the NC91 throughout the discharge process. The reduced electrochemical polarization and improved Li^+ diffusion coefficients are ascribed from $LiAlO_2$ coating and Al^{3+} doping. The Al^{3+} doping can reduce Li/Ni disorder and increase the lattice parameters, which can significantly boost Li^+ transfer inside the lattice. Besides, the coating of $LiAlO_2$ with high Li^+ conductivity is capable for improving Li^+ transfer at the interface.

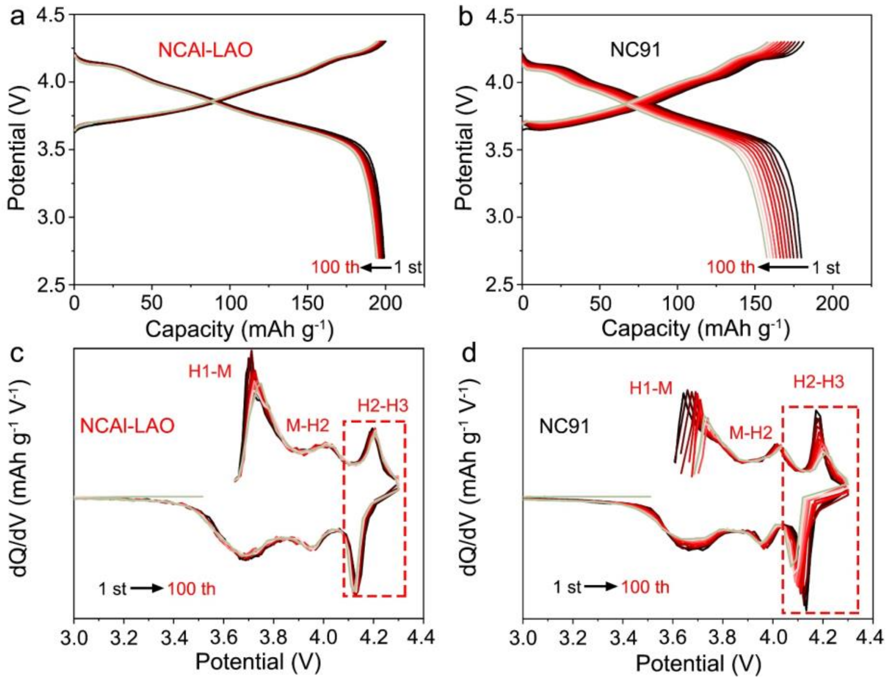

Supplementary Fig. 14 The charge/discharge curves of (a) NCAl-LAO and (b) NC91 from 1 st to 100 th cycles. dQ dV^{-1} profiles obtained by differentiating charge-discharge curves at different cycle numbers of (c) NCAl-LAO and (d) NC91.

The charge/discharge profiles of both NCAl-LAO and NC91 over 100 cycles were depicted in **Supplementary Fig. 14a, b**. At the initial cycles, both samples display the similar curves with the distinguishable voltage plateaus. After 100 cycles, the characteristic voltage plateaus referred to H2-H3 phase transition at 4.15 V can retain well for the NCAl-LAO. Instead, the voltage plateaus of NC91 are gradually disappeared with the increase of cycle numbers, indicating serious structure degradation. To meticulously investigate the evolution of phase transition throughout the cycle, the dQ dV^{-1} profiles were collected by differentiating the charging/discharging curves of NCAl-LAO and NC91 (**Supplementary Fig. 14c, d**). Both cathodes undergo a series of phase transitions, which is in accord with CV curves. The intensities of the peaks related to phase transition for the NCAl-LAO, especially the H2-H3 phase transition, hardly changed after 100 cycles, indicating the excellent reversibility. In

comparison, the peaks of the NC91 noticeably drop in intensity and become gradually polarized, which is derived from the structural degradation during long-term cycling.

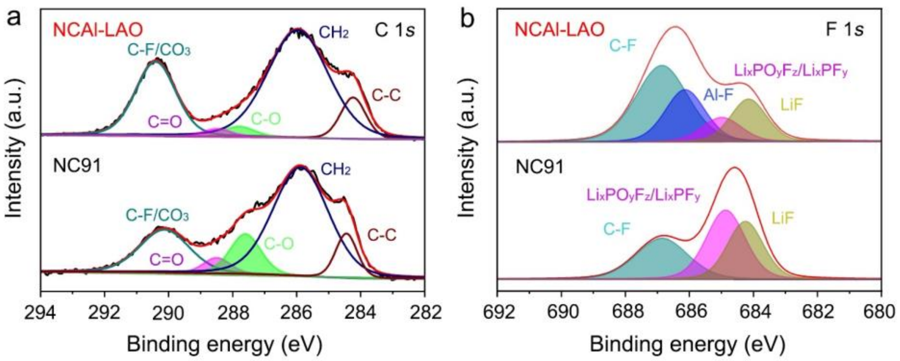

Supplementary Fig. 15 (a) C 1*s* and (b) F 1*s* XPS spectra of NCAl-LAO and NC91 after 100 cycles.

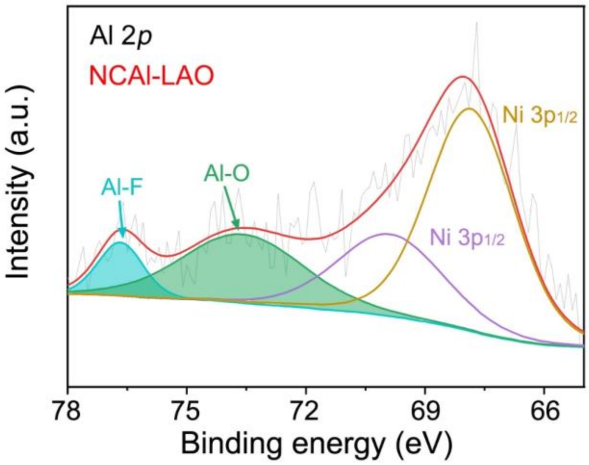

Supplementary Fig. 16 Al 2*p* XPS spectra of NCAl-LAO after 100 cycles.

The XPS surface analysis was performed for NCAl-LAO and NC91 after 100 cycles at 1 C to certify the stability of interfacial chemistry, and the intensities of all the XPS spectra were normalized. As depicted in C 1*s* peaks (**Supplementary Fig. 15a**), the peaks at ca 285 eV represent the Super P (C-C), while the peaks of C-H and C-F located at ca 286 and ca 290 eV are both related to PVDF binder. Meanwhile, the peaks at ca 287 eV and 288 eV can be

designated to ether and carbonate, which are attributed from the electrolyte decomposition[8]. **Supplementary Fig. 15b** shows the F 1*s* spectra for both samples, the C-F bonds (687 eV) are assigned to the PVDF binder. Meanwhile, the $Li_xPO_yF_z/Li_xPF_y$ (685 eV) and LiF (684 eV) peaks are the components of CEI films derived from the parasitic reactions at the electrode/electrolyte interface[9]. It is noteworthy that the peak intensities of carbon-oxygen compounds, $Li_xPO_yF_z/Li_xPF_y$ and LiF in NCAl-LAO are all lower than that of NC91, suggesting less parasitic reactions and thinner cathode-electrolyte interphase (CEI) films. Therefore, the analysis of the interface chemical composition has validated that the interface stability has indeed be improved after modification. One side, the $LiAlO_2$ coating can refrain the direct contact between the cathode and the electrolyte, forming more stable and thinner CEI films. On the other hand, the improvement of structural stability can reduce the release of oxidizing species (O^{2-}, O^-, etc.) during phase transformation, which can mitigate the decomposition of electrolyte to generate impurity (such as HF). Furthermore, the existence of Al-F signal in F 1*s* region (ca 686.5 eV in **Supplementary Fig. 15b**) and Al 2*p* region (ca 76.5 eV in **Supplementary Fig. 16**) of NCAl-LAO after cycling indicate that the $LiAlO_2$ layer can be partly fluorinated to form AlF_3 during electrochemical process[10]. A similar phenomenon was also observed when using another Al-containg coating[11,12]. According to previous studies[13,14], this fluorination can effectively scavenge HF in electrolyte and the resultant AlF_3 can promote the formation of stable cathode-electrolyte interface (CEI) films, which are beneficial for inhibiting dissolution of transiation metals.

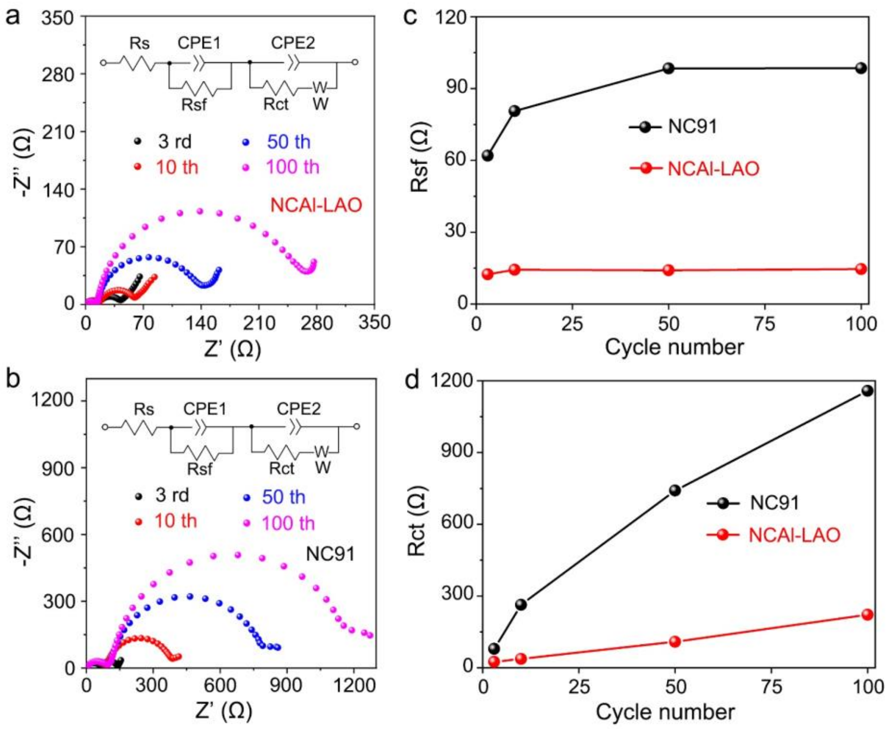

Supplementary Fig. 17 (a, b) Nyquist plots after different cycles of NCAl-LAO and NC91, and the insets show the equivalent circuits for the impedance spectra. (c, d) Change of R_{sf} and R_{ct} for NCAl-LAO and NC91 with respect to the cycle number.

The electrochemical impedance measurements of NCAl-LAO and NC91 were performed after various cycles. The Nyquist plots and corresponding equivalent circuits are shown in **Supplementary Fig. 17a, b**. The first semicircle in the high-medium frequency range corresponds to the surface film resistances (R_{sf}), resulting from the CEI films, while the second semicircle at medium-low frequency is identified as the charge-transfer resistance (R_{ct}) of the cathode[15]. The data extracted from the Nyquist plots with respect to the cycle number are displayed in **Supplementary Fig. 17c, d** and **Supplementary Table 7**. The R_{sf} of NCAl-LAO is 13.2 Ω at 3rd cycle and almost unchanged with the increase of the cycle numbers, whereas the R_{sf} of NC91 is 69.8 Ω at 3rd cycle and gradually enhances to 102.2 Ω at 100th cycle. The coating of $LiAlO_2$ layer can mitigate parasitic reactions at the interface to reduce the thickness

of the cathode-electrolyte interface (CEI) films and meanwhile improve Li^+ conductivity at electrode/electrolyte interface benefitted from high Li^+ conductivity of $LiAlO_2$ (up to $3*10^{-5}$ Ω^{-1} cm^{-1}). Therefore, R_{sf} is reduced after modification. Furthermore, the improved structure stability and particle integrity by the surface $LiAlO_2$ coating and bulk Al^{3+} doping can alleviate the formation of new exposed surface during cycle process, and thus inhibit the formation of new CEI films. Compared to the obvious R_{sf} increase of pristine NC91 during cycling, the R_{sf} of NCAl-LAO has almost no change. The Rct of NCAl-LAO and NC91 at 3rd cycle are 30.1 and 75.0 Ω, respectively, and the values both increase as cycling proceeded, which is caused by the deterioration of crystal structure. Surprisingly, the R_{ct} of NCAl-LAO increases to 258.2 Ω at 100th cycle, which is much lower than that of NC91 (1141.2 Ω). The impedance changes during uninterrupted charging/discharging process certify the enhanced interfacial and structural stability

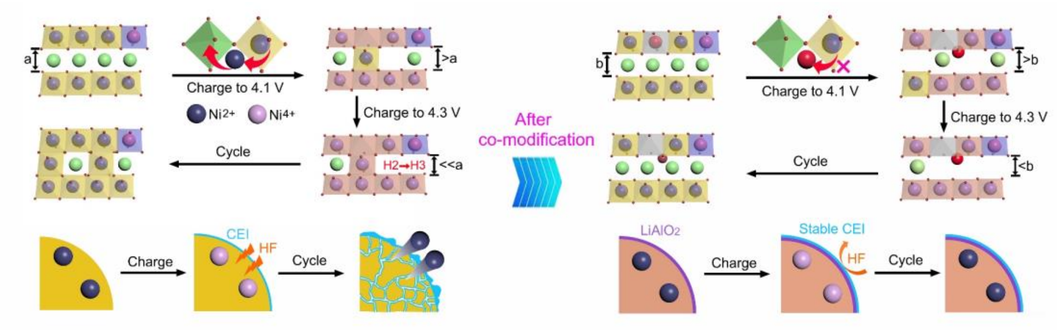

Supplementary Fig. 18 Schematic illustration of the superiority of modification strategy for stabilizing lattice structure and interface chemical.

The schematic illustration vividly exhibits the superiority of the Ni-rich cathode after oxalate-assisted modification strategy. The mitigated Ni^{2+} transfer during the electrochemical process could restrain the transformation of the layered phase to the electrochemical inert spinel and

rock-salt phases. In addition, the coating of $LiAlO_2$ as a physical protection barrier can also block the detrimental parasitic reactions and Ni^{2+} dissolution at the electrode-electrolyte interface.

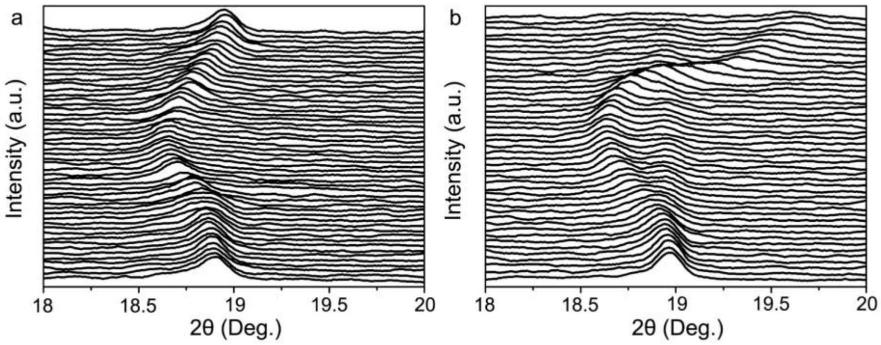

Supplementary Fig. 19 The (003) peaks at *in-situ* XRD patterns of the (a) NCAl-LAO and (b) NC91.

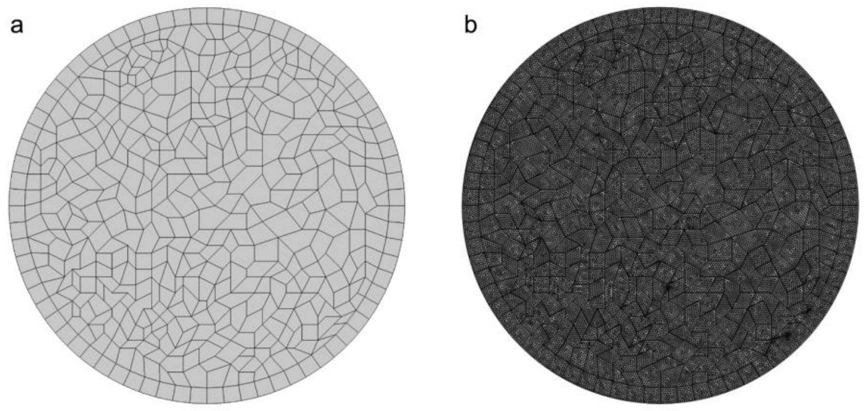

Supplementary Fig. 20 (a) 2D model and (b) mesh for finite element analysis

The simulation model was established according to the microstructure characteristics obtained from SEM and TEM images. As shown in **Supplementary Fig. 20a**, the sizes of primary particles were set as 200-500 nm and the constituent secondary particle displayed a diameter of 10 μm. It should be pointed out that the primary particles were considered as single crystals and the homologous crystallographic orientations were stochastically set. The mesh for this model

was displayed in **Supplementary Fig. 20b** and the simulations with disregarded any plastic deformation were performed by using mechanics and thermal modules in 2D finite element analysis.

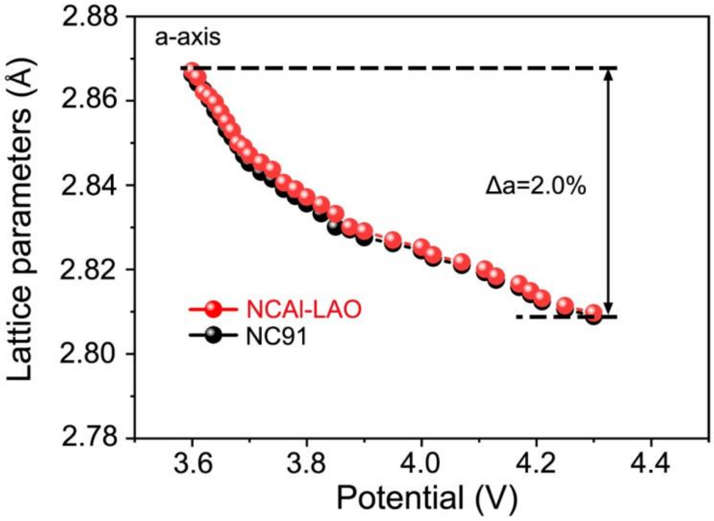

Supplementary Fig. 21 The variations of a-axis lattice parameters as a function of the charging voltage.

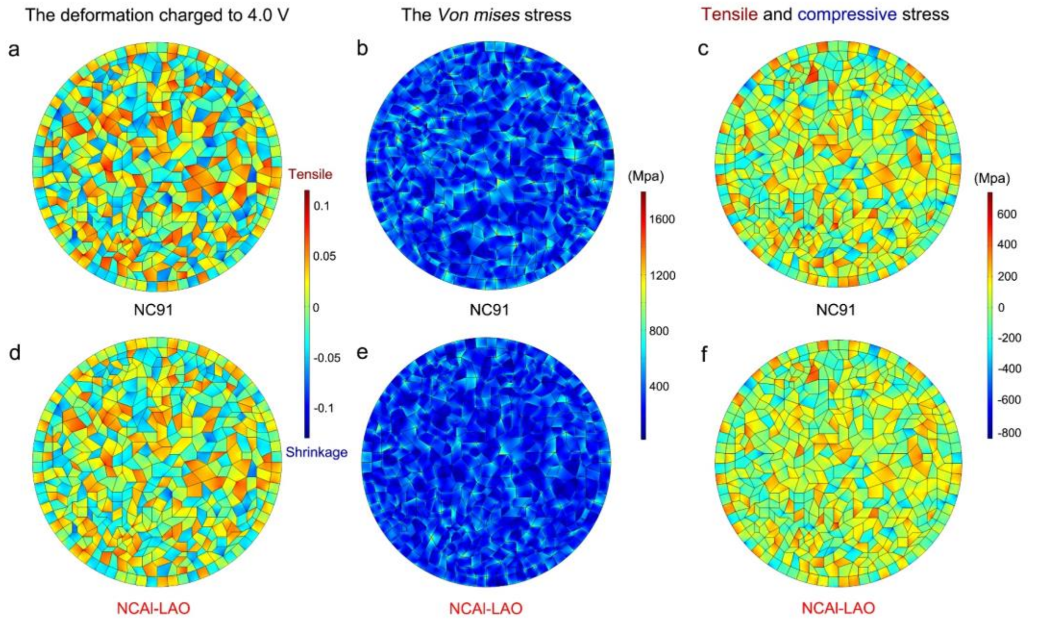

Supplementary Fig. 22 The distribution of (a) volume deformation, (b) *Von mises* stress, (c) tensile and compressive stress thoughout the secondary particles for NC91 and NCAl-LAO when charging to 4.0 V.

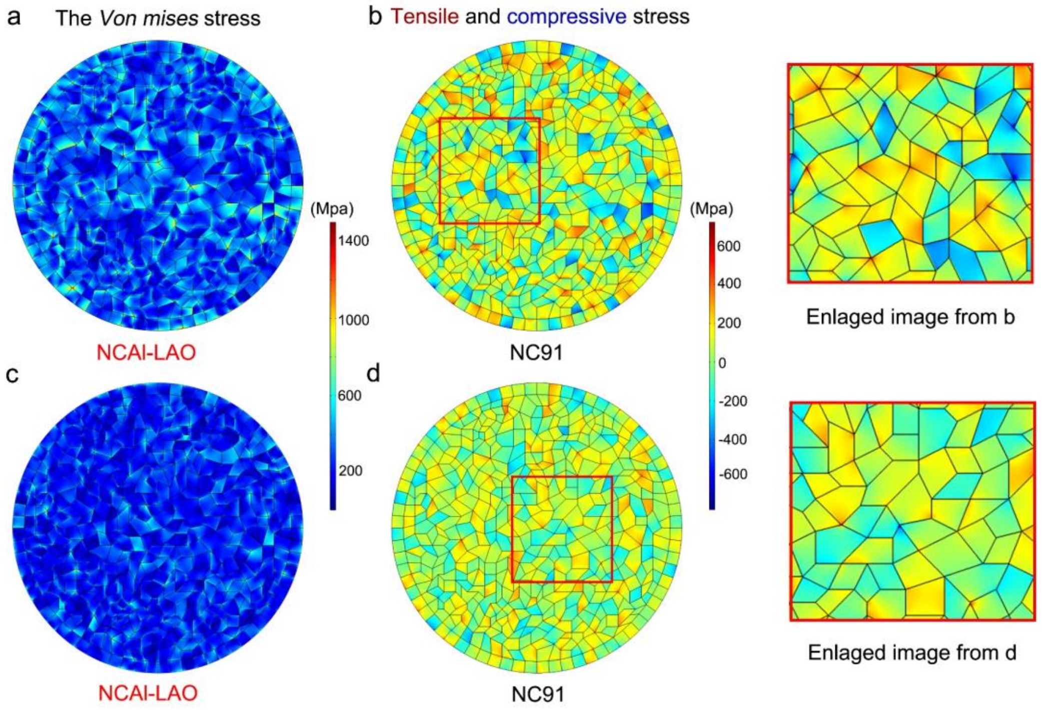

Supplementary Fig. 23 The distribution of (a, c) *Von mises* stress, (b, d) tensile and compressive stress thoughout the secondary particels for NC91 and NCAl-LAO when charging to 4.3 V.

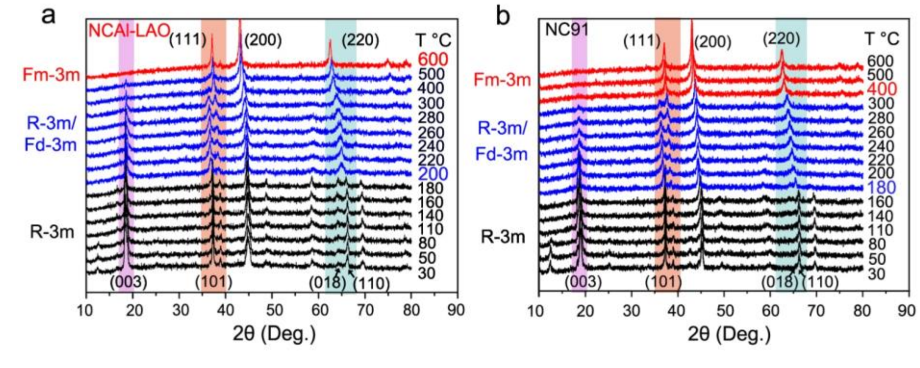

Supplementary Fig. 24 Full range HT-XRD patterns of delithiated NCAl-LAO and NC91 cathodes (charged to 4.3 V) during heating from 30 to 600 °C.

Supplementary Tables

Supplementary Table 1 Formation energies of Al atom substituting at Li and Ni sites.

Doping site	ΔE (eV/f.u.)	$E_{doped\ system}$ (eV)	$E_{pristine\ system}$ (eV)	$\mu_{substituted\ atom}$ (eV)	$\mu_{doped\ atom}$ (eV)
Al→Li	-0.57	-546.698	-539.611	-1.898	-3.741
Al→Ni	-0.61	-547.216	-539.611	-1.637	-3.741

Supplementary Table 2 Migration energies of Al and Ni atom from O_{TM} to T_{Li} site.

Migrating style	Migrating ion	ΔE (eV/f.u.)	$E_{migrated\ system}$ (eV)	$E_{pristine\ system}$ (eV)
O_{TM}→T_{Li}	Al	0.19	-386.953	-388.665
	Ni	0.33	-385.656	-388.665

Supplementary Table 3 Formation energies of Al and Ni atom at T_{Li} site.

Doping site	ΔE (eV/f.u.)	$E_{doped\ system}$ (eV)	$E_{pristine\ system}$ (eV)	$\mu_{substituted\ atom}$ (eV)	$\mu_{doped\ atom}$ (eV)
Al→T_{Li}	-1.06	-393.398	-380.066	\	-3.741
Al→T_{Ni}	-0.70	-387.967	-380.066	\	-1.637

Supplementary Table 4 Chemical compositions of Ni, Co, and Al for NC91, NCAl-LAO-L, NCAl-LAO, and NCAl-LAO-H measured by ICP-AES.

Sample	Chemical composition		
	Ni	Co	Al
NC	0.896	0.104	-
NCAl-LAO-L	0.889	0.099	0.012
NCAl-LAO	0.868	0.096	0.036

NCAl-LAO-H	0.849	0.094	0.057

Supplementary Table 5 The values of $I_{(003)}/I_{(104)}$ for NC91, NCAl-LAO-L, NCAl-LAO, and NCAl-LAO-H.

Samples	NC	NCAl-LAO-L	NCAl-LAO	NCAl-LAO-H
I(003)/I(104)	1.51	1.65	1.83	2.29

Supplementary Table 6 Rietveld refinement results of XRD data for NC91 and NCAl-LAO.

Sample	a (Å)	c (Å)	Volume ($Å^3$)	Site		x	y	z	Occupancy	R_{wp}	χ^2
NC91	2.8667	14.1707	100.786	Li	3a	0	0	0	0.975(3)	8.78%	1.09
				Li	3b	0	0	0.5	0.024(7)		
				Ni	3b	0	0	0.5	0.871(3)		
				Ni	3a	0	0	0	0.024(7)		
				Co	3b	0	0	0.5	0.104		
				O	6a	0	0	0.255	1		
NCAl-LAO	2.8671	14.1842	100.957	Li	3a	0	0	0	0.986(5)	9.42%	1.03
				Li	3b	0	0	0.5	0.013(5)		
				Ni	3b	0	0	0.5	0.864(0)		
				Ni	3a	0	0	0	0.013(5)		
				Co	3b	0	0	0.5	0.0975		
				Al	3b	0	0	0.5	0.025		
				O	6a	0	0	0.255	1		

Supplementary Table 7 The R_{sf} and R_{ct} data extracted from the Nyquist plots with respect to cycle number

Samples	NC91		NCAl-LAO	
	R_{sf}	R_{ct}	R_{sf}	R_{ct}
3 rd	62.0	79.1	12.4	24.8
10 th	80.6	263.7	14.3	37.4
50 th	98.4	741.0	14.0	108.9
100 th	98.6	1158.0	14.4	222.1

Reference

[1] Li, H., Zhou, P., Liu, F., Li, H., Cheng, F. & Chen, J. Stabilizing nickel-rich layered oxide cathodes by magnesium doping for rechargeable lithium-ion batteries. *Chem. Sci.* **10**, 1374-1379 (2019).

[2] Wu, Y. et al. Enhancing the Li-ion storage performance of graphite anode material modified by $LiAlO_2$. *Electrochim. Acta* **235**, 463-470 (2017).

[3] Yu, H., Li, Y., Hu, Y., Jiang, H. & Li, C. Concurrently coating and doping high-valence vanadium in nickel-rich lithiated oxides for high-rate and stable lithium-ion batteries. *Ind. Eng. Chem. Res.* **58**, 4108-4115 (2019).

[4] Sun, H., Choi, W., Lee, J., Oh, I. & Jung, H. Control of electrochemical properties of nickel-rich layered cathode materials for lithium ion batteries by variation of the manganese to cobalt ratio. *J. Power Sources* **275**, 877-883 (2015).

[5] Jiang, Q., Yu, H., Hu, Y., Jiang, H. & Li, C. Exposed surface engineering of high-voltage $LiNi_{0.5}Co_{0.2}Mn_{0.3}O_2$ cathode materials enables high-rate and durable Li-ion batteries. *Ind. Eng. Chem. Res.* **58**, 23099-23105 (2019).

[6] Deng, Z. et al. 3D ordered macroporous MoS_2@C nanostructure for flexible Li-ion batteries. *Adv. Mater.* **29**, 1603020 (2017).

[7] Amin, R., Ravnsbæk, D. & Chiang, Y. Characterization of electronic and ionic transport in $Li_{1-x}Ni_{0.8}Co_{0.15}Al_{0.05}O_2$ (NCA). *J. Electrochem. Soc.* **162**, 1163-1169 (2015).

[8] Li, Y. et al. A novel electrolyte salt additive for lithium-ion batteries with voltages greater than 4.7 V. *Adv. Energy Mater.* **7**, 1601397 (2017).

[9] Xu, M. et al. Development of novel lithium borate additives for designed surface modification of high voltage ($LiNi_{0.5}Mn_{1.5}O_4$) cathode. *Energy Environ. Sci.* **9**, 1308-1319 (2016).

[10] Xie, J. et al. Atomic layer deposition of stable $LiAlF_4$ lithium ion conductive interfacial layer for stable cathode cycling. *ACS Nano* **11**, 7019-7027 (2017).

[11] Myung, S. et al. Functionality of oxide coating for $Li[Li_{0.05}Ni_{0.4}Co_{0.15}Mn_{0.4}]O_2$ as positive electrode materials for lithium-ion secondary batteries. *J. Phys. Chem. C* **111**, 4061-4067 (2007).

[12] Myung, S., Izumi, K., Komaba, S., Sun, Y., Yashiro, H. & Kumagai, N. Role of alumina coating on Li-Ni-Co-Mn-O particles as positive electrode material for lithium-ion batteries. *Chem. Mater.* **17**, 3695-3704 (2005).

[13] Hall, D., Gauthier, R., Eldesoky, A., Murray, V. & Dahn, J. New chemical insights into the beneficial role of Al_2O_3 cathode coatings in lithium-ion cells. *ACS Appl. Mater. Interfaces* **11**, 14095-14100 (2019).

[14] Sun, Y., Lee, M., Yoon, C., Hassoun, J., Amine, K. & Scrosati, B. The role of AlF_3 coatings in improving electrochemical cycling of Li-enriched nickel-manganese oxide electrodes for Li-ion batteries. *Adv. Mater.* **24**, 1192-1196 (2012).

[15] Li, Y., Yu, H., Hu, Y., Jiang, H. & Li, C. Surface-engineering of layered $LiNi_{0.815}Co_{0.15}Al_{0.035}O_2$ cathode material for high-energy and stable Li-ion batteries. *J. Energy Chem.* **27**, 559-564 (2018).
